# Identification of the Roles of a Stemness Index Based on mRNA Expression in the Prognosis and Metabolic Reprograming of Pancreatic Ductal Adenocarcinoma

**DOI:** 10.3389/fonc.2021.643465

**Published:** 2021-04-12

**Authors:** Rong Tang, Xiaomeng Liu, Wei Wang, Jie Hua, Jin Xu, Chen Liang, Qingcai Meng, Jiang Liu, Bo Zhang, Xianjun Yu, Si Shi

**Affiliations:** ^1^ Department of Pancreatic Surgery, Fudan University Shanghai Cancer Center, Shanghai, China; ^2^ Department of Oncology, Shanghai Medical College, Fudan University, Shanghai, China; ^3^ Shanghai Pancreatic Cancer Institute, Shanghai, China; ^4^ Pancreatic Cancer Institute, Fudan University, Shanghai, China

**Keywords:** pancreatic cancer, stemness, metabolic rewiring, metabolism, transcriptome

## Abstract

**Background:**

Cancer stem cells (CSCs) are widely thought to contribute to the dismal prognosis of pancreatic ductal adenocarcinoma (PDAC). CSCs share biological features with adult stem cells, such as longevity, self-renewal capacity, differentiation, drug resistance, and the requirement for a niche; these features play a decisive role in cancer progression. A prominent characteristic of PDAC is metabolic reprogramming, which provides sufficient nutrients to support rapid tumor cell growth. However, whether PDAC stemness is correlated with metabolic reprogramming remains unknown.

**Method:**

RNA sequencing data of PDAC, including read counts and fragments per kilobase of transcript per million mapped reads (FPKM), were collected from The Cancer Genome Atlas-Pancreatic Adenocarcinoma (TCGA-PAAD) database. Single-sample gene set enrichment analysis (GSEA) was used to calculate the relative activities of metabolic pathways in each PDAC sample. Quantitative real-time PCR was performed to validate the expression levels of genes of interest.

**Results:**

The overall survival (OS) of patients with high mRNA expression-based stemness index (mRNAsi) values was significantly worse than that of their counterparts with low mRNAsi values (*P* = 0.003). This survival disadvantage was independent of baseline clinical characteristics. Gene ontology (GO) analysis, Kyoto Encyclopedia of Genes and Genomes (KEGG) analysis and GSEA showed that the differentially expressed genes between patients with high and low mRNAsi values were mainly enriched in oncogenic and metabolic pathways. Weighted gene coexpression network analysis (WGCNA) revealed 8 independent gene modules that were significantly associated with mRNAsi and 12 metabolic pathways. Unsupervised clustering based on the key genes in each module identified two PDAC subgroups characterized by different mRNAsi values and metabolic activities. Univariate Cox regression analysis identified 14 genes beneficial to OS from 95 key genes selected from the eight independent gene modules from WGCNA. Among them, MAGEH1, MAP3K3, and PODN were downregulated in both pancreatic tissues and cell lines.

**Conclusion:**

The present study showed that PDAC samples with high mRNAsi values exhibited aberrant activation of multiple metabolic pathways, and the patients from whom these samples were obtained had a poor prognosis. Future studies are expected to investigate the underlying mechanism based on the crosstalk between PDAC stemness and metabolic rewiring.

## Introduction

Pancreatic ductal adenocarcinoma (PDAC) is one of the most lethal malignancies, with mortality rates almost equal to its incidence (1). In recent decades, the incidence of pancreatic cancer has increased annually, imposing heavy health and economic burdens on many countries (2). Radical surgical resection remains the most efficient treatment method for PDAC (3). Due to the limited understanding of the evolution of its genetic characteristics and the concealed anatomical position of the pancreas, it is difficult to detect PDAC early, which would prevent metastasis and avoid the need for radical treatment (4–6). Many researchers believe that cancer stem cells (CSCs) may contribute to the dismal prognosis of PDAC (7). CSCs share biological features with adult stem cells, such as longevity, capacity for self-renewal, differentiation, drug resistance and the requirement for a niche, features that play a decisive role in cancer progression (8). Malta et al. identified stemness features associated with oncogenic dedifferentiation in a pan-cancer profile using a machine learning algorithm (9). They introduced stem cell indices to evaluate the stemness of each tumor sample in The Cancer Genome Atlas (TCGA) database and found that such indices could accurately predict metastatic events and interpret intratumoral heterogeneity. In particular, the mRNA expression-based stemness index (mRNAsi) reflects cancer stemness by analyzing transcriptomic data of cancer samples; signatures for cancer stemness are derived from the pre-analysis of normal stem cells and their progeny using one-class logistic regression.

A prominent characteristic of PDAC is metabolic rewiring, which provides sufficient nutrients to support rapid tumor cell growth (10, 11). Driven by oncogene-mediated cell-autonomous pathways and the unique physiology of the tumor microenvironment, PDAC cell metabolism is extensively reprogrammed, including increased glycolysis and glutamine metabolism. Our previous work revealed that different SMAD4 mutation statuses dictate various metabolic preferences in PDAC (12). Hence, targeting tumor metabolism may be an efficient weapon to curb tumor progression (13).

CSCs possess unique metabolic plasticity, allowing them to rapidly respond and adapt to environmental disturbances (14). The metabolism of CSCs is thought to be tumor type-specific: some tumors (such as nasopharyngeal and liver cancers) rely on a glycolytic program, whereas others (such as lung, glioma or colon cancer) use oxidative phosphorylation, suggesting that metabolic plasticity confers on CSCs the ability to adapt to challenges from the environment and support self-renewal (15). Nonetheless, the correlation between stemness and metabolic landscapes in PDAC has yet to be systematically analyzed.

Here, we performed a bioinformatics analysis to systematically investigate whether the stemness of PDAC affects patient prognosis and intratumoral metabolic rewiring.

## Methods

### Data Source and Selection

RNA sequencing (RNA-seq) data, including read counts and fragments per kilobase of transcript per million mapped reads (FPKM), were collected from The Cancer Genome Atlas-Pancreatic Adenocarcinoma (TCGA-PAAD). According to the annotation of TCGA-PAAD, we excluded nonductal-derived tumors and normal adjacent samples. Only PDAC samples remained for subsequent bioinformatics analysis. Clinical data, such as overall survival (OS), were also downloaded from the abovementioned data sets. Two GEO data sets, GSE11838 and GSE32676, were included to validate the expression of key genes between tumor and adjacent tissues. The stem cell indices based on the transcriptome of each PDAC sample were acquired from a previously reported study (9) and referred to as the mRNA expression-based stemness index (mRNAsi) in the following sections. Gene sets involved in arginine and proline metabolism; glycine, serine, and threonine metabolism; branched chain amino acid catabolism; glycolysis; glutamate and glutamine metabolism; glutathione synthesis and recycling; phospholipid metabolism; gluconeogenesis; oxidative phosphorylation; pentose phosphate pathway; lipolysis in adipose tissue; and triglyceride biosynthesis were downloaded from the Molecular Signatures Database (MSigDB) (https://www.gsea-msigdb.org/gsea/msigdb/index.jsp). The genes involved in each pathway are presented in **Supplementary Table 1**.

### Mutation Analysis

Whole-exome sequencing data were downloaded from TCGA-PAAD-VarScan. The gene mutation distribution and abundance were visualized using the “maftools” R package.

### Gene Ontology (GO) and KOBAS-Kyoto Encyclopedia of Genes and Genomes (KEGG) Pathway Analyses

Differentially expressed genes (DEGs) between the mRNAsi_high and mRNAsi_low groups were detected using the Wilcoxon test with the “limma” R package (version 3.4). The cutoff values to define the DEGs were log [fold change (FC)] > 2 and false discovery rate (FDR) < 0.05. GO functional enrichment analysis and KEGG pathway enrichment analysis of DEGs were performed by the “clusterProfiler,” “org.Hs.eg.db,” “plot,” and “ggplot2” R packages. Gene set enrichment analysis (GSEA) was also performed to explore the functions of the DEGs using the “clusterProfiler,” “org.Hs.eg.db,” “enrichplot,” and “limma” R packages.

### Single-Sample GSEA (ssGSEA)

Based on the transcriptomes of PDAC samples and metabolism-related gene sets, we performed ssGSEA to calculate the activity of each metabolic pathway in every PDAC sample. The R packages “GSEABase” and “GSVA” were used to conduct ssGSEA.

### Weighted Gene Coexpression Network Analysis (WGCNA)

WGCNA was performed using the WGCNA R package (16). According to the WGCNA manual, the coexpression of genes in the pan-gene landscape was analyzed instead of only DEGs. Initially, RNA-seq data were filtered to exclude outliers. The coexpression similarity matrix consisted of the absolute values of the correlations between transcript expression levels. A Pearson correlation matrix was constructed for paired genes. Then, we constructed a weighted adjacency matrix using the power function amn = |cmn|β, where cmn = the Pearson correlation between gene m and gene n and amn = the adjacency between gene m and gene n. The β value emphasizes strong correlations between genes and penalizes weak correlations. Next, an appropriate β value was taken to increase the similarity matrix and achieve a scale-free coexpression network. The adjacency matrix was then converted into a topological overlap matrix (TOM), which reflects the network connectivity of genes defined as the sum of adjacent genes generated by other networks. Average linkage hierarchical clustering was further conducted based on TOM-based dissimilarity measurements, and the minimum size (genome) of the gene dendrogram was 30. Through further analysis of modules, we calculated their dissimilarity and constructed module dendrograms.

To evaluate the significance of each module, gene significance (GS) was calculated to reflect the correlations between genes and sample traits. Module eigengenes (MEs) were considered the major components in the principal component analysis of each gene module, and the expression patterns of all genes were summarized as a single feature expression profile within a given module. Next, GS was determined by the log10 conversion of the *P* value in the linear regression of gene expression and clinical data (GS = log_10_ *P*). Module significance (MS) was the average GS within the module and was calculated to measure the correlation between the module and sample traits. Statistical significance was determined using the relevant *P* values. To increase the capacity of the modules, we selected a cutoff (<0.25) to merge some modules with similar heights. The resulting gene models contained genes that were highly correlated and might exert identical biological functions or have associations with the same phenotypes.

To identify key genes in each module, we calculated GS and module membership (MM, correlation between the module’s own genes and gene expression profiles) for each key gene and set their thresholds. The thresholds for screening key genes in the module were defined as cor. gene MM > 0.8 and cor. gene GS > 0.5.

### Survival Analysis

PDAC patients were assigned to two groups based on the median of each stem cell index. A Kaplan-Meier curve was depicted to present the variation in the OS rate along with an increased follow-up period. A log-rank test was conducted to evaluate the difference between two groups in terms of survival expectancy.

Univariate Cox regression analysis was conducted to assess whether the selected key genes were correlated with patient survival. *P* < 0.05 was considered statistically significant.

### Cell Culture and qRT-PCR

The human pancreatic cancer cell lines Capan-1, Panc-1, Mia-paca2, CF-PAC1, and BxPC-3 were obtained from the American Type Culture Collection. The human pancreatic ductal cell line HPDE was also obtained from the American Type Culture Collection. Capan-1 and CF-PAC1 cells were cultured in Iscove’s modified Dulbecco’s medium (IMDM) with 10% fetal bovine serum. Panc-1, BxPC-3, Mia-paca2, and HPDE cells were cultured in Dulbecco’s modified Eagle’s medium (DMEM) with 10% fetal bovine serum. CAFs were first separated and purified from human pancreatic cancer tissues in our laboratory based on the study by Walter et al. and then subjected to immortalization treatment (17). Fresh pancreatic cancer tissue was minced into 1–3 mm^3^ fragments and digested with 0.25% trypsin at 37°C for 30 min. The resulting fragments were centrifuged at 600*g* for 5 min and washed once with Dulbecco’s modified Eagle’s medium (DMEM) containing 10% fetal bovine serum (FBS). The tissue fragments were then plated and allowed to adhere. After incubation at 37°C for several days, fibroblast outgrowth from the tissue fragments occurred. Fibroblasts were sub-cultured by trypsinization for two to three passages until free of epithelial cell contamination and maintained in DMEM supplemented with 10% FBS, 2% penicillin and streptomycin (Invitrogen). Cells were grown at 37°C in a humidified atmosphere containing 5% CO_2_. CAFs were cultured in DMEM with 10% fetal bovine serum. Quantitative real-time PCR was performed as described previously (12). All reactions were run in triplicate, and the primer sequences are listed in **Supplementary Table 2**. RNA was extracted from 43 pairs of resected pancreatic cancer tissues and adjacent normal tissues preserved in RNAlater using the SteadyPure Universal RNA Extraction Kit (AG21017). The paired t test was performed to assess the statistical significance of the differential expression between tumor and adjacent normal tissues.

## Results

### Lower mRNAsi Predicts Prolonged OS in Patients With Pancreatic Cancer

**Figure 1:**
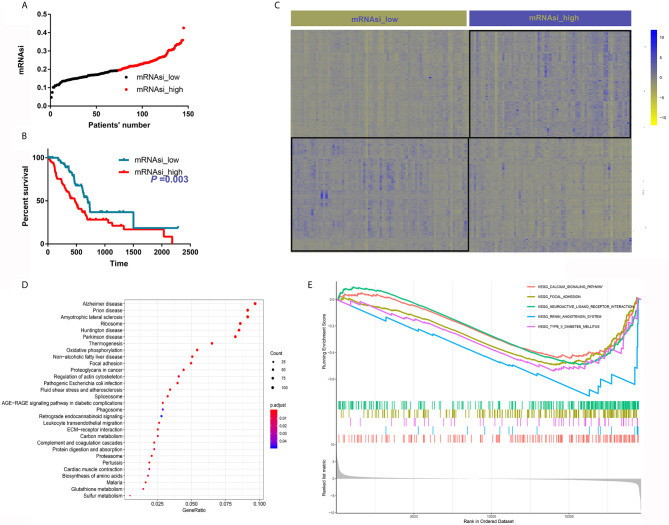
mRNAsi is associated with PDAC patient survival and oncogenic and metabolic pathways. **(A)** Patients were divided into two groups based on the median mRNAsi. **(B)** High mRNAsi values predicted poor OS of patients with PDAC. **(C)** DEGs between samples with high and low mRNAsi values. **(D)** KEGG analysis of the DEGs. **(E)** The top five pathways upregulated in samples with high mRNAsi values.

A total of 145 PDAC samples with complete follow-up data were divided into two groups based on the median mRNAsi (**Figure 1A**). Then, we compared the baseline clinical data between the two groups; the distribution of sex, age, race, liver metastasis, history of chronic pancreatitis, residual tumor, number of lymph nodes, location, T stage, N stage, and American Joint Committee on Cancer (AJCC) grade were comparable between the two groups (**Table 1**). Next, we depicted the survival curve for each group and compared differences in OS using the log-rank test. The OS of patients with high mRNAsi values was significantly worse than that of their counterparts with low mRNAsi values (*P* = 0.003) (**Figure 1B**).

**Table 1:** Basic characteristics of patients with different mRNAsi values.

	mRNAsi_low (n = 72)	mRNAsi_high (n = 73)	Significance
Sex (male)	52.80%	56.20%	*P* = 0.682
Age (year)	63.64	66.45	*P* = 0.121
Race (white)	88.90%	86.30%	*P* = 0.637
Liver metastasis (yes)	25.00%	17.81%	*P* = 0.291
History of chronic pancreatitis (yes)	8.33%	9.59%	*P* = 0.791
Residual tumor (R0)	56.90%	54.80%	*P* = 0.794
Number of lymph nodes (median)	2	2	*P* = 0.780
Location(head)	81.94%	78.08%	*P* = 0.561
T stage (T3/4)	81.94%	87.67%	*P* = 0.337
N stage (N1)	79.17%	68.49%	*P* = 0.144
AJCC (>2b)	80.56%	68.49%	*P* = 0.096
Grade	45.83%	47.95%	*P* = 0.799

### GO and KEGG Analyses of DEGs Between Patients With High and Low mRNAsi Values

A total of 2116 DEGs were identified with the criteria logFC>2 and adjusted *P* value<0.05 (**Figure 1C**). GO analysis suggested that these DEGs were highly enriched for functions associated with cancer initiation and progression, such as focal adhesion, collagen-containing extracellular matrix and substantially activated RNA transcription and protein translation (**Supplementary Figure 1**). KEGG analysis showed that these DEGs were enriched in some oncogenic mechanisms, such as focal adhesion and were also enriched in many metabolic pathways, such as oxidative phosphorylation, carbon metabolism, glutathione metabolism and biosynthesis of amino acids (**Figure 1D**). GSEA was also performed, and calcium signaling and focal adhesion were the two most enriched signaling pathways (**Figure 1E**).

### PDAC Samples With High and Low mRNAsi Values Have Different Mutational Landscapes and Match Different Previously Reported Molecular Subtypes

**Figure 2:**
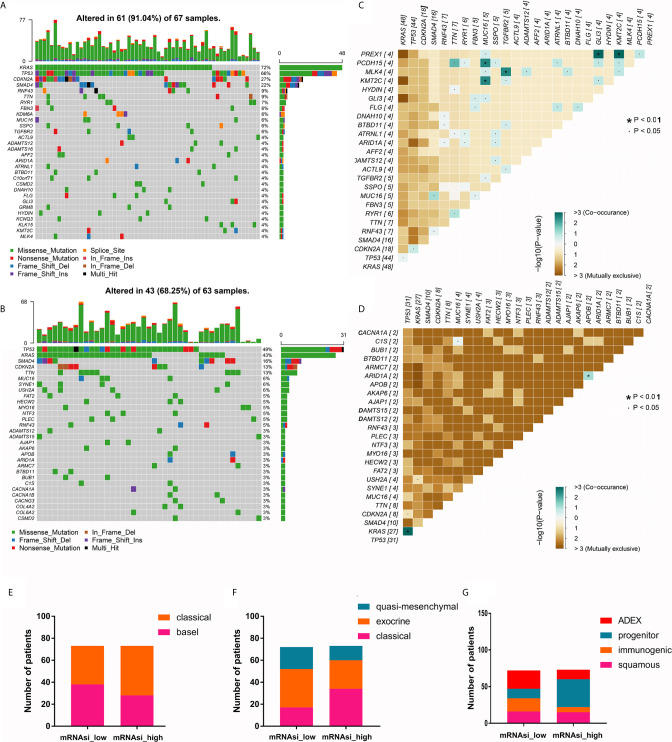
The mutational landscape and molecular subtypes of samples with high and low mRNAsi values. **(A, B)** The top 30 most mutated genes in PDAC samples with high and low mRNAsi values. **(C, D)** Cooccurrence and mutual exclusion of mutated genes in PDAC samples with high and low mRNAsi values. **(E–G)** Proportions of PDAC samples of different molecular subtypes (Moffitt cluster, Collison cluster and Bailey cluster) with high and low mRNAsi values.

We further compared the differences in mutation profiles between patients with high and low mRNAsi values. The top 30 most frequently mutated genes in the two groups are shown in **Figures 2A, B**. Notably, these genes were mutated in 91.04% of PDAC samples with high mRNAsi values but only 68.25% of samples with low mRNAsi values, suggesting that the frequencies of these mutations increased with mRNAsi elevation. In addition, as expected, KRAS was the most frequently mutated gene in samples with high mRNAsi values; however, the most mutated gene in the low- mRNAsi group was TP53. Another important finding was that the cooccurrence of gene mutations was more common in samples with high mRNAsi values than in samples with low mRNAsi values (**Figures 2C, D** **)**. The mutation landscape profiles of the two groups are shown in **Supplementary Figure 2**.

Next, we analyzed the proportions of patients with the three previously reported molecular subtypes with high and low mRNAsi values (**Table 2** **)**. We found a significant inconsistency between different subtypes of the Collisson and Bailey clusters in PDAC samples with high and low mRNAsi values (*P* = 0.0145 and *P* = 0.0001, respectively). Specifically, the proportion of the classic subtype in the Collisson cluster was larger among high-mRNAsi samples than among low-mRNAsi samples; similarly, the percentage of the progenitor subtype in the Bailey cluster was also larger in high-mRNAsi samples (**Figures 2E–G**).

**Table 2:** The distribution of patients with previously reported molecular subtypes between the different mRNAsi groups.

	mRNAsi_low(n=72)	mRNAsi_high(n=73)	
**Moffitt clusters**			
Basel	38	28	
Classical	35	45	*P* = 0.096
**Collisson clusters**			
Classical	17	34	
Exocrine	35	26	
Quasi-mesenchymal	20	13	*P* = 0.0145
**Bailey Clusters**			
Squamous	16	15	
Immunogenic	18	7	
Progenitor	13	38	
ADEX	25	13	*P* = 0.0001

We also compared differences in the immune microenvironment between the high and low mRNAsi groups. Differences in the infiltration of a many types of immune were observed between the high and low mRNAsi groups. Notably, low-mRNAsi samples were enriched in fibroblasts and M2-polarized macrophages, whereas high-mRNAsi samples were enriched in activated CD4+ T cells (**Supplementary Figure 3**).

### Identification of Eight Independent Gene Modules Associated With mRNAsi and Metabolic Reprogramming in Pancreatic Cancer by WGCNA

**Figure 3:**
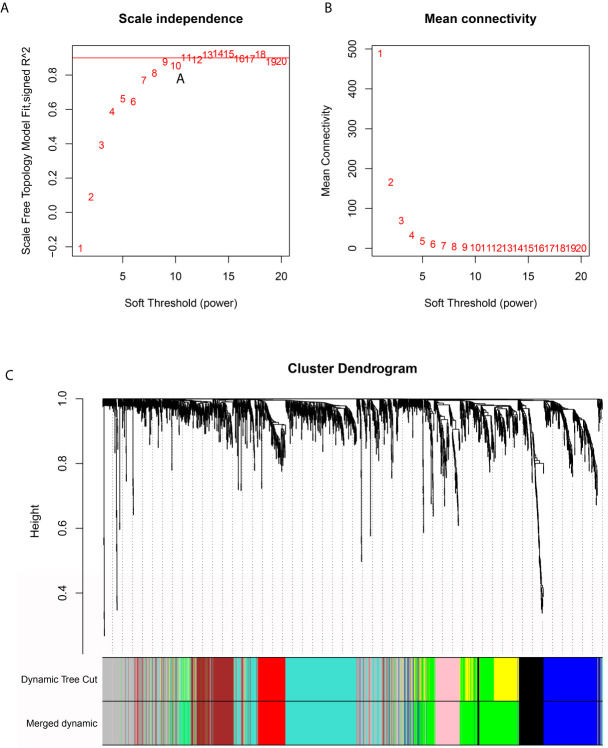
WGCNA-based identification of 8 independent gene modules based on PDAC transcriptome data. **(A, B)** An appropriate β value was selected to increase the similarity matrix and obtain a scale-free coexpression network. **(C)** Eight gene modules remained after adjacent gene modules with high similarity were merged.

The RNA-seq data were first filtered to exclude outliers (**Supplementary Figure 4A**). Then, we visualized the associations between mRNAsi and metabolic activities in the PDAC samples (**Supplementary Figure 4B**). Next, an appropriate β value (0.9) was identified to increase the similarity matrix and obtain a scale-free coexpression network (**Figures 3A, B** **)**. Average linkage hierarchical clustering was further conducted based on topological overlap matrix (TOM)-based dissimilarity measurements, and the minimum size (genome) of the gene dendrogram was 30 (**Supplementary Figures 4C, D**). To reduce the number of gene modules, dynamic tree cuts with high similarities were merged based on a cutoff height of (0.25) (**Supplementary Figure 4E**), and the distribution of genes in every module was visualized as shown in **Figure 3C**. Finally, eight independent gene modules were identified.

**Figure 4:**
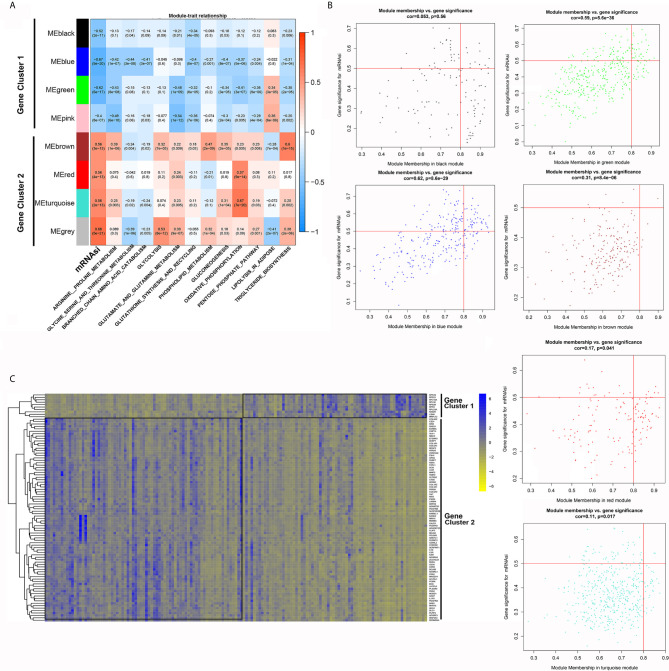
WGCNA-based identification of gene modules and key genes associated with PDAC stemness. **(A)** Module-trait relationships revealed the correlations among gene modules, mRNAsi and metabolic pathways. **(B)** Identification of key genes significantly associated with both mRNAsi and modules in each gene module. **(C)** The differential expression of the selected key genes between the mRNAsi_high and mRNAsi_low groups.

We presented the module-trait relationships by showing the correlations among eight clustered gene modules, mRNAsi and the activity of each metabolic pathway (**Figure 4A**). The eight gene modules were further divided into two gene clusters: gene cluster 1 was inversely associated with mRNAsi and most metabolic pathways except for lipolysis activity, while gene cluster 2 was positively correlated with mRNAsi and lipolysis activity.

Then, we investigated the key genes that were highly associated with mRNAsi in each module by setting the threshold parameters as MM > 0.8 and GS > 0.5. A total of 95 genes in six modules met these criteria (**Figure 4B**). Among them, ten genes belonged to the modules in gene cluster 2, while the others belonged to the modules in gene cluster 1 (**Figure 4C**).

### Unsupervised Clustering Based on the Key Genes Identified in Each Module

**Figure 5:**
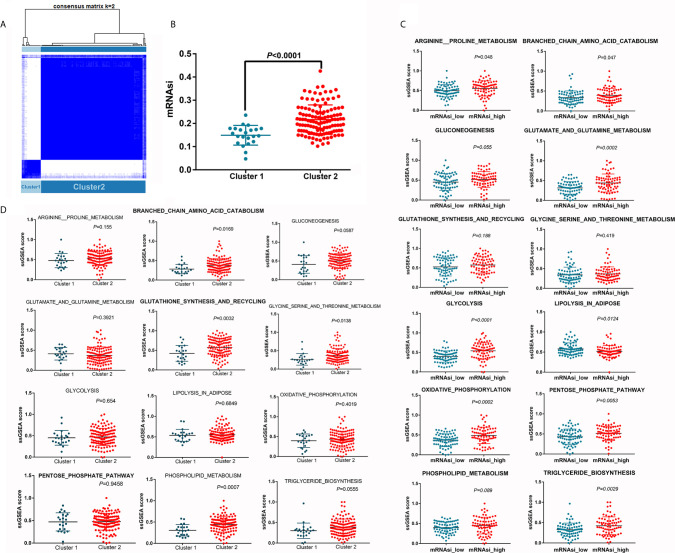
Differences in metabolic pathway activity between different clusters. **(A)** Unsupervised clustering distinguished two clusters based on the selected key genes. **(B)** mRNAsi is increased in cluster 2. **(C)** Differences in metabolic pathway activity between the mRNAsi_high and mRNAsi_low groups. **(D)** Differences in metabolic pathway activity between clusters 1 and 2.

Based on the expression levels of the 95 selected key genes, the PDAC samples were clustered into two groups (referred to as cluster 1 and cluster 2) (**Figure 5A**). Samples in cluster 2 were characterized by significantly higher mRNAsi values (**Figure 5B**). We further investigated the activity of each metabolic pathway between high and low mRNAsi samples. The activities of arginine and proline metabolism, branched chain amino acid catabolism, glutamate and glutamine metabolism, glycolysis, oxidative phosphorylation, pentose phosphate pathway, and triglyceride biosynthesis were significantly upregulated in PDAC samples with high mRNAsi values compared with samples with low mRNAsi values, whereas lipolysis activity decreased with increasing mRNAsi value (**Figure 5C**). In addition, the activities of branched chain amino acid catabolism; glutathione synthesis and recycling; glycine, serine and threonine metabolism; and phospholipid metabolism were increased in cluster 2 (**Figure 5D**). Hence, the activity of branched chain amino acid catabolism was increased in both cluster 2 and in samples with high mRNAsi values, suggesting that the key genes used to define cluster 2 could partially explain the concurrently increased mRNAsi values and branched chain amino acid catabolism.

### In Vitro Validation of Differentially Expressed Prognosis-Related Genes USsing qRT-PCR in Pancreatic Cancer Cell Lines, CAFs and HPDE Cells

**Figure 6:**
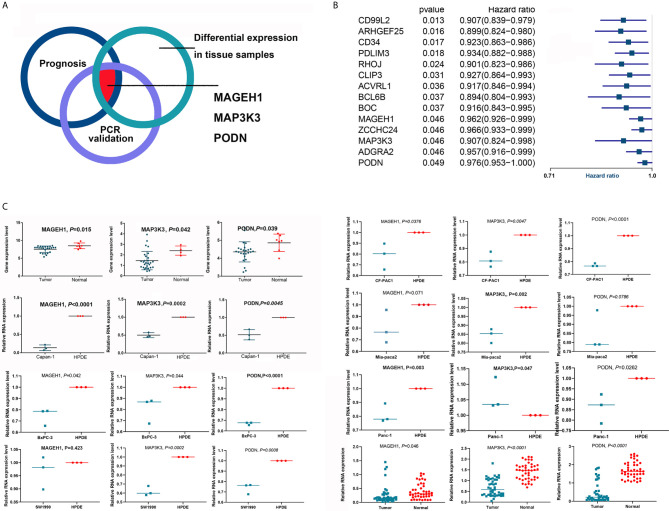
Validation of the differential expression of key OS-related genes. **(A)** The diagram shows that MAGEH1, MAP3K3 and PODN were validated in silico and by qRT-PCR. **(B)** Fourteen key genes were associated with the OS of patients with PDAC. **(C)** MAGEH1, MAP3K3 and PODN were downregulated in tumor tissues and cell lines.

Due to the heterogeneity of tumor tissue, differential gene expression may occur only in some cell types and not throughout the entire tumor. We used qRT-PCR to validate the expression of the genes of interest (**Figure 6A**) in pancreatic cancer cell lines and normal pancreatic ductal cells (HPDE). A total of 14 of 95 key genes were found to be associated with the OS of patients with PDAC. Interestingly, all 14 genes were predicted to favor survival (**Figure 6B**) and were expected to be downregulated in tumor tissues and cells. Therefore, if a specific gene was downregulated in tumor tissues (GSE11838 or GSE32676), we further validated the expression of this gene using qRT-PCR in vitro. MAGEH1 and PODN were downregulated in PDAC tissues in GSE32676, and MAGEH1 was downregulated in PDAC tissues in GSE11838. These in silico results were validated using 43 pairs of resected pancreatic cancer samples, which also showed that the expression levels of MAGEH1, MAP3K3 and PODN were downregulated in tumor tissues. The relative mRNA expression levels of these genes were also decreased in most pancreatic cancer cell lines compared with HPDE cells (**Figure 6C**). In addition, the expression levels of these genes were comparable between CAFs and HPDEs (**Supplementary Figure 5**).

## Discussion

Tumor heterogeneity is an ongoing challenge for cancer therapy (18). Many studies have demonstrated that tumors harbor subclones that differ with respect to karyotype and chemotherapy sensitivity (19, 20), and CSCs are thought to be one of the determining factors of intratumor heterogeneity. Recently, Wang et al. identified tetraspanin CD9 as a marker of PDAC tumor-initiating cells (21). Cells with high CD9 expression have increased organoid formation capability and generate tumor grafts more easily in vivo. Tumors originating from cells with high CD9 expression recapitulate the cellular heterogeneity of primary PDAC, whereas cells with low CD9 expression produce only duct-like epithelial progeny. Interestingly, this study also illustrated that CD9 promotes plasma membrane localization of the glutamine transporter ASCT2 and further increases uptake of glutamine, an essential metabolite for cancer progression, in PDAC cells.

In fact, metabolic rewiring is a widely acknowledged phenomenon in cancer development, and several metabolic pathways are aberrantly activated intratumorally, such as glycolysis and glutamine metabolism (13, 22). A recent study uncovered the robust dependence of PDAC on cysteine metabolism. Through upregulated uptake of cysteine from the tumor microenvironment, PDAC cells can instantly clear lipid peroxides and evade ferroptosis, a potent nonapoptotic cell death mechanism (23). Similarly, another study reported that genetic ablation of the cystine transporter in PDAC cells inhibits mTORC1, proliferation and tumor formation via nutrient and oxidative stresses (24).

In this context, systematically investigating the association and crosstalk between PDAC stemness and metabolic reprogramming is significant but experimentally challenging using traditional methods. Advances in next-generation sequencing and RNA-seq have provided useful tools to investigate the correlations between different gene signatures (25, 26). The activity of a specific signaling pathway can be estimated by related gene expression using the algorithm “GSVA” (27). PDAC stemness can be estimated using transcriptome data through one-class logistic regression as innovated by Malta et al. (9). In the present study, we first revealed that a higher mRNAsi predicts worse OS in PDAC patients, suggesting that increased stemness is associated with a dismal prognosis of PDAC. Then, we identified 8 independent gene modules based on PDAC transcriptome data by WGCNA and analyzed their associations with mRNAsi and various metabolic pathways. Ninety-five key genes tightly associated with both gene modules and stemness were selected, and their prognostic implications in PDAC were also established. Finally, MAGEH1, PODN, and MAP3K3 were identified as three mRNAsi-related tumor suppressor genes that were downregulated in tumor tissues and whose overexpression was associated with prolonged OS. Several previous studies have reported roles of these genes in other types of cancer. For example, Wang et al. suggested that MAGEH1 expression is downregulated in HCC tumor tissues (28). MAGEH1 reduces HCC cell proliferation, migration and invasion abilities. Low MAGEH1 expression is significantly correlated with poor prognosis in HCC patients (28). Few studies have reported the role of PODN in cancer. Bai et al. recently reported that PODN is hypermethylated and could be used to predict patient survival in gastric cancer (29). MAP3K3 seems to play a dual role in cancer. Some studies have shown that it acts as a tumor suppressor that inhibits Hedgehog pathway-dependent medulloblastoma, and its overexpression in tumor cells and tumor-infiltrating lymphocytes is correlated with favorable lung cancer patient survival (30, 31). Some other studies have implied that MAP3K3 is a protumor molecule (32, 33).

This study has notable strengths. First, few previous studies have systematically investigated the associations among stemness, prognosis and metabolic rewiring in PDAC. Second, most of the clinical characteristics were comparable between the two groups with high and low mRNAsi values, and the sample size of our study was relatively large, which ensured the reliability of our results. However, this study also has some limitations. External validation based on transcriptome, proteome and metabolome analyses using fresh PDAC samples is needed to further confirm our conclusions. Such studies are hindered by the difficulty of collecting PDAC samples and the high cost of sequencing. In addition, we revealed only the association between stemness and metabolic rewiring in PDAC, and the causality between them was not elucidated. Furthermore, a common weakness of GO and KEGG analyses is that they do not reveal which differentially expressed pathways are enriched in which group. Hence, we provide the differentially expressed genes between the high- and low-mRNAsi groups as **Supplementary Table 3**. More basic studies are warranted to determine whether CSCs can induce intratumoral metabolic rewiring or metabolite alteration in the tumor microenvironment to promote PDAC stemness.

In conclusion, the present study showed that high mRNAsi values were associated with a poor prognosis of PDAC patients and the aberrant activation of multiple metabolic pathways in PDAC samples. Future studies are expected to investigate the underlying mechanism based on the crosstalk between PDAC stemness and metabolic rewiring.

## Data Availability Statement

The datasets presented in this study can be found in online repositories. The names of the repository/repositories and accession number(s) can be found in the article/**Supplementary Material**.

## Author Contributions

RT and XL performed the bioinformatics analysis. WW and JH were responsible for the statistical analysis. JX, CL, and JL revised the tables and figures. BZ, XY, and SS designed the study. All authors contributed to the article and approved the submitted version.

## Funding

This work was supported in part by the National Natural Science Foundation of China (81802352), the National Science Foundation for Distinguished Young Scholars of China (81625016), the Scientific Innovation Project of Shanghai Education Committee (2019-01-07-00-07-E00057), and the Clinical and Scientific Innovation Project of Shanghai Hospital Development Center (SHDC12018109).

## Conflict of Interest

The authors declare that the research was conducted in the absence of any commercial or financial relationships that could be construed as a potential conflict of interest.

## Supplementary Material

Supplementary Figure 1 GO analysis of DEGs between PDAC samples with high and low mRNAsi values.

Supplementary Figure 2 Summary of mutation information from patients with high and low mRNAsi values.

Supplementary Figure 3 Differences in immune microenvironment between samples with high and low mRNAsi values.

Supplementary Figure 4 The process of WGCNA. **(A)** RNA-seq data were filtered to exclude outliers. **(B)** The corresponding associations among PDAC samples, their mRNAsi and metabolic activities were determined. **(C, D)** Average linkage hierarchical clustering was further conducted based on TOM-based dissimilarity measurements. **(E)** To reduce the number of gene modules, dynamic tree cuts with high similarities were merged based on a cutoff height of 0.25.

Supplementary Figure 5 The expression of target genes in fibroblasts derived from pancreatic cancer.
