# Supplementary figures and images for: Identification of the Roles of a Stemness Index Based on mRNA Expression in the Prognosis and Metabolic Reprograming of Pancreatic Ductal Adenocarcinoma
Source: Front Oncol. 2021 Apr 12;11:643465. doi: 10.3389/fonc.2021.643465 (PMC8071957; doi:10.3389/fonc.2021.643465)

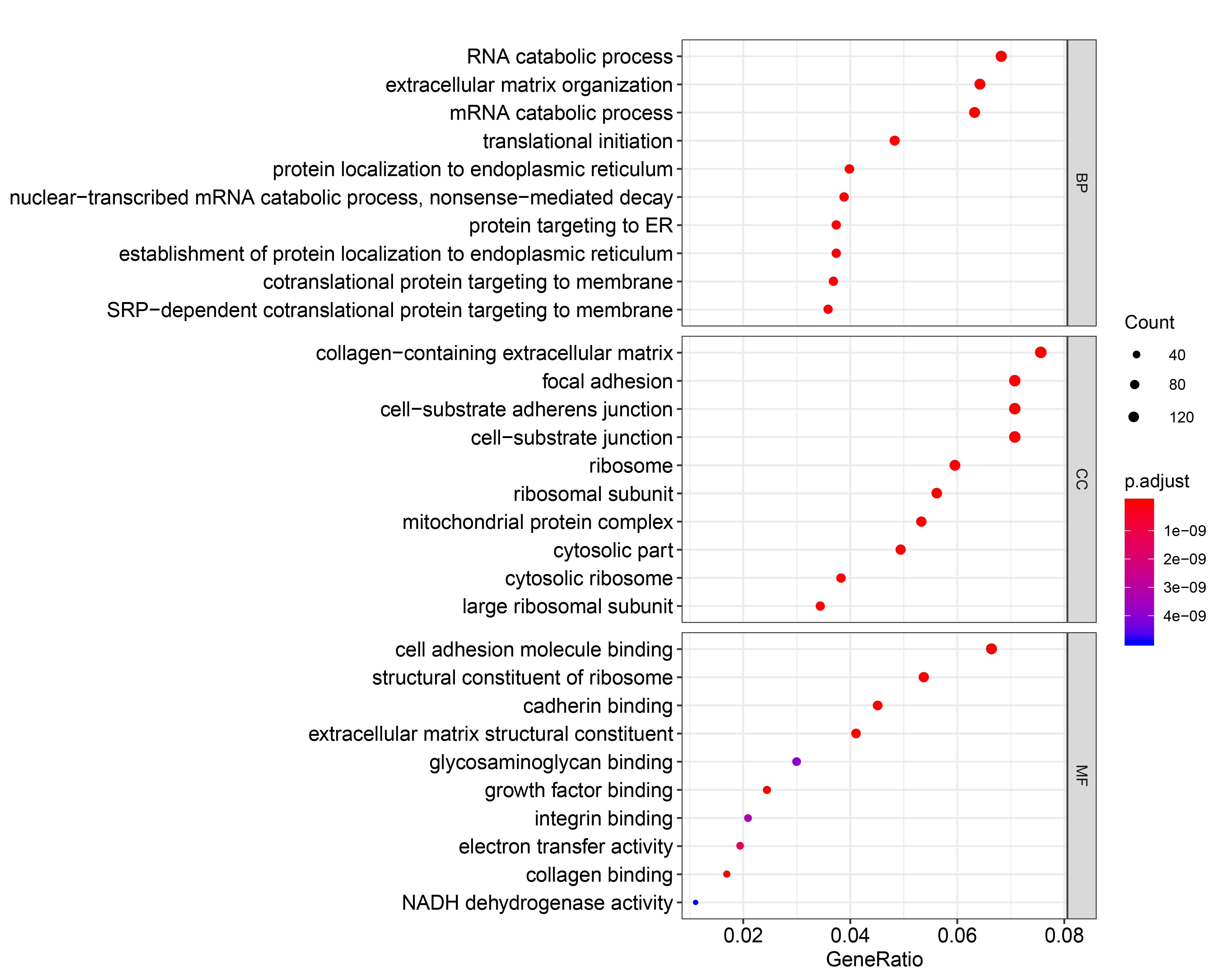

Supplement: Supplementary Figure 1 — GO analysis of DEGs between PDAC samples with high and low mRNAsi values. [file Image_1.jpeg]

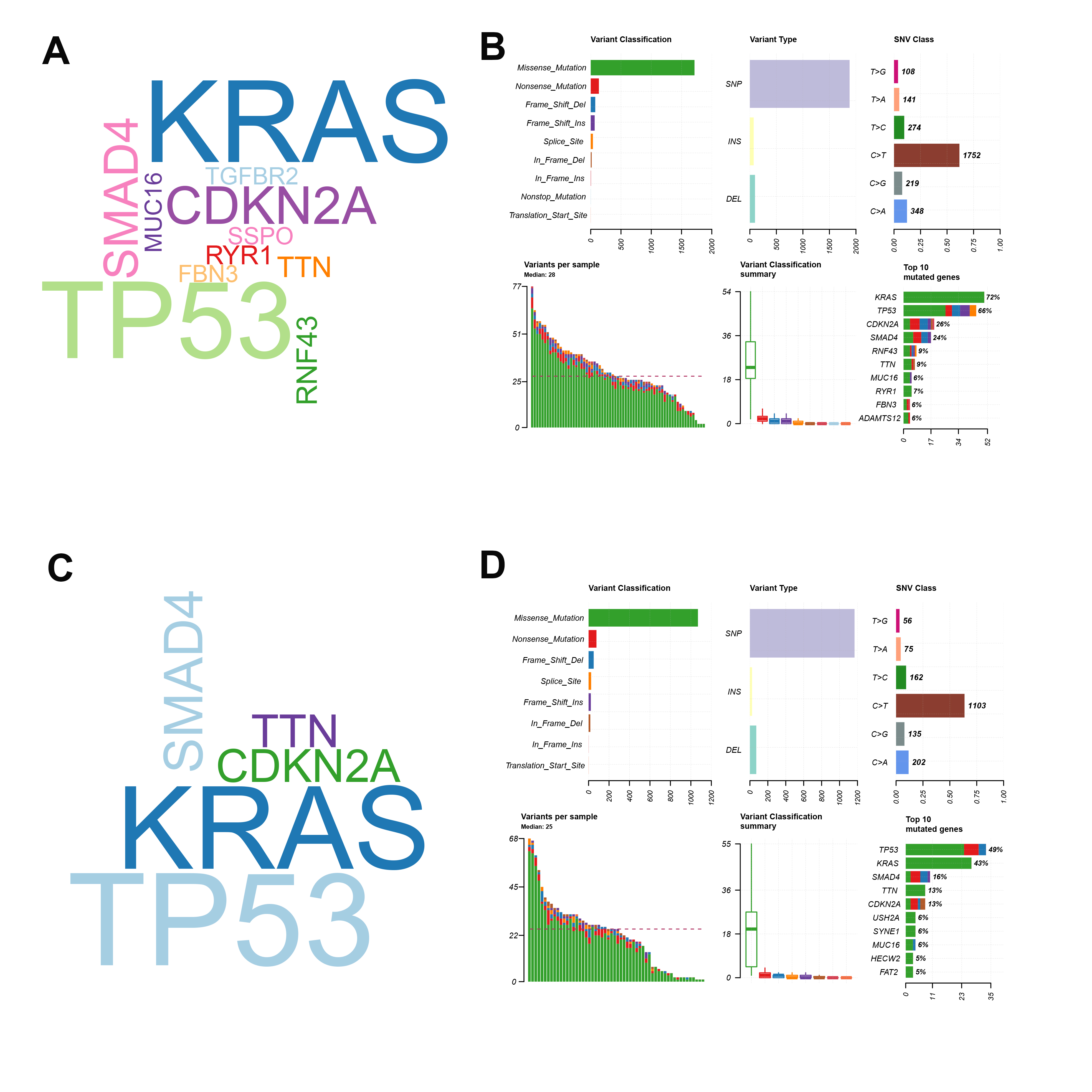

Supplement: Supplementary Figure 2 — Summary of mutation information from patients with high and low mRNAsi values. [file Image_2.jpeg]

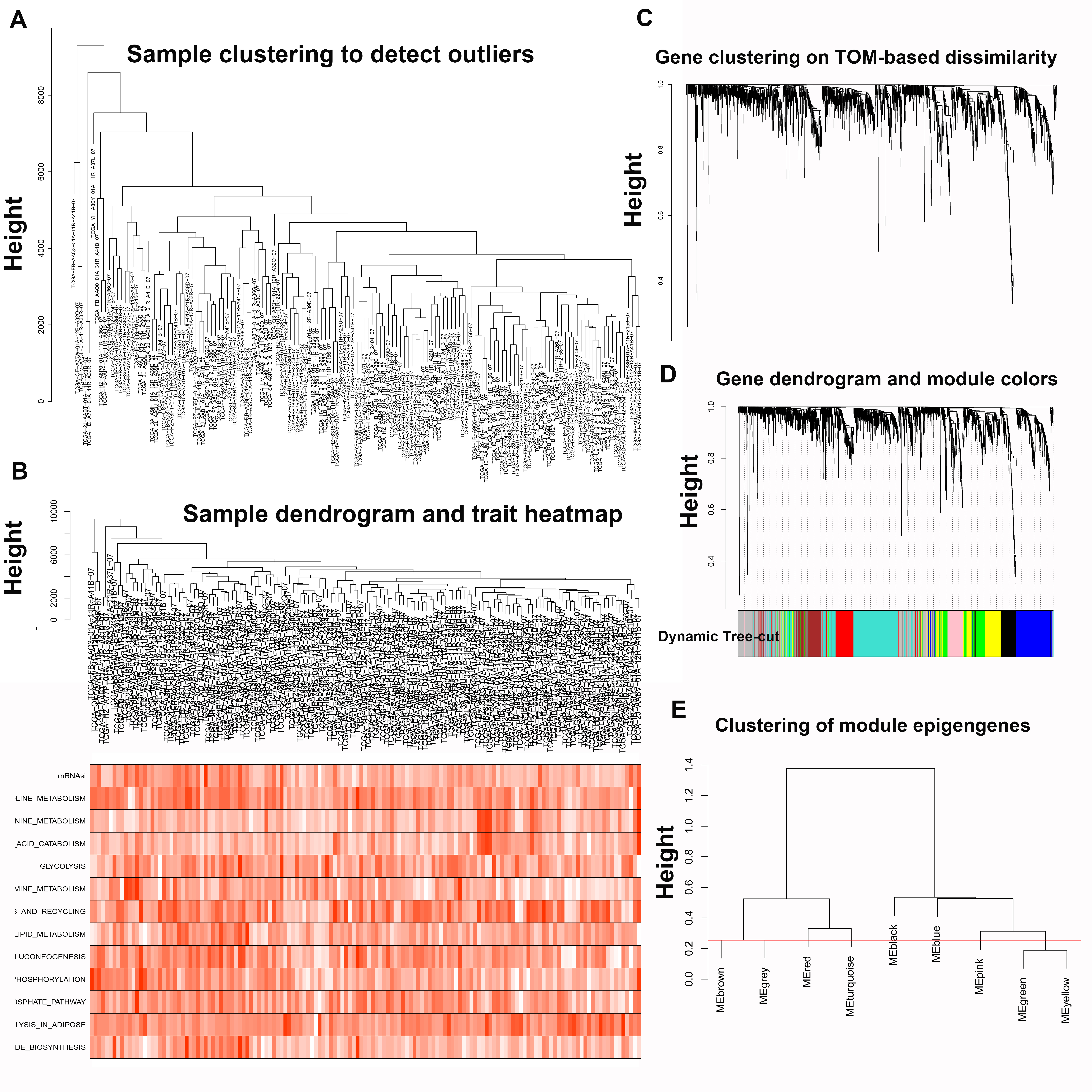

Supplement: Supplementary Figure 3 — Differences in immune microenvironment between samples with high and low mRNAsi values. [file Image_3.jpeg]

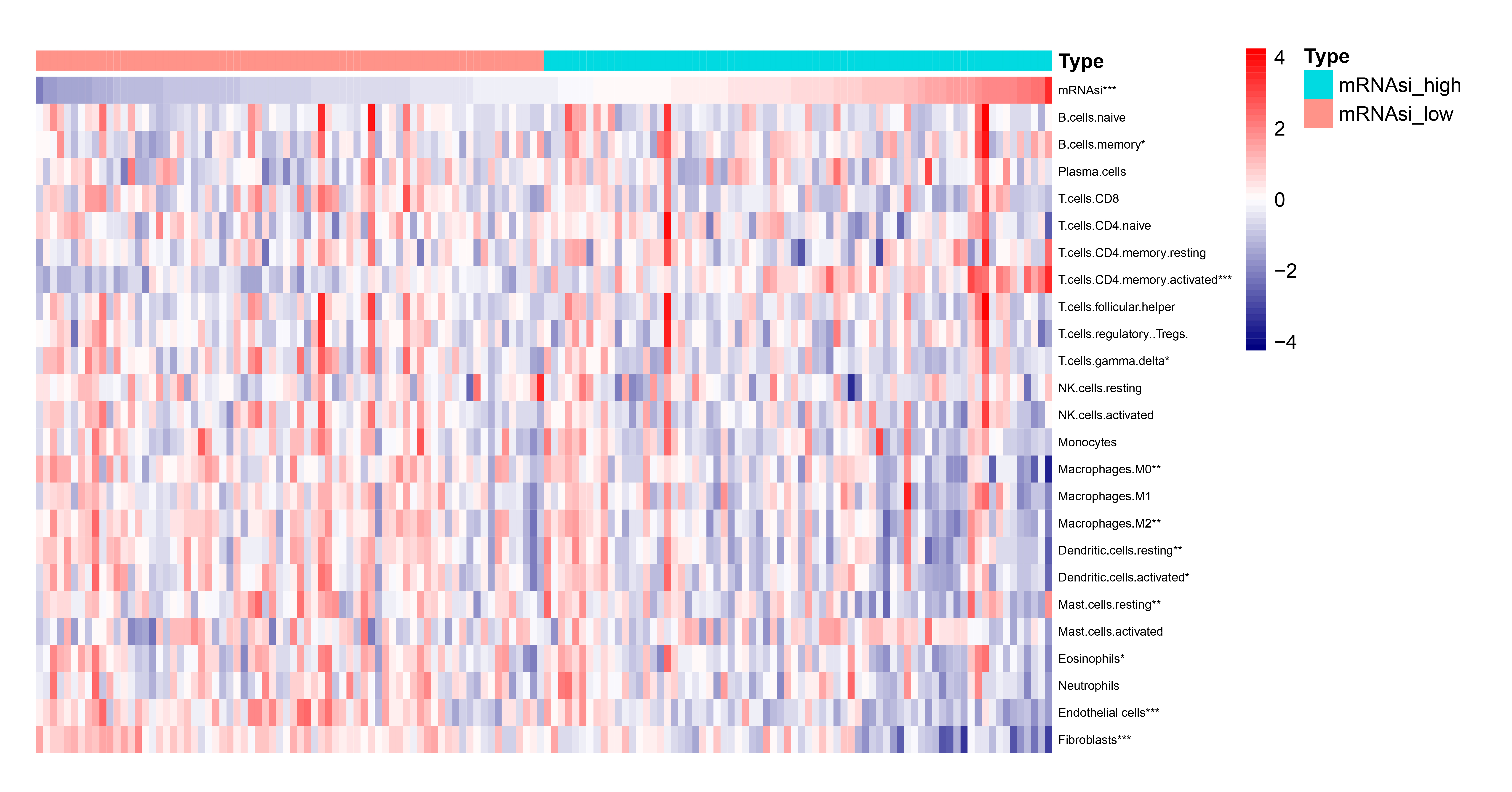

Supplement: Supplementary Figure 4 — The process of WGCNA. (A) RNA-seq data were filtered to exclude outliers. (B) The corresponding associations among PDAC samples, their mRNAsi and metabolic activities were determined. (C, D) Average linkage hierarchical clustering was further conducted based on TOM-based dissimilarity measurements. (E) To reduce the number of gene modules, dynamic tree cuts with high similarities were merged based on a cutoff height of 0.25. [file Image_4.jpeg]

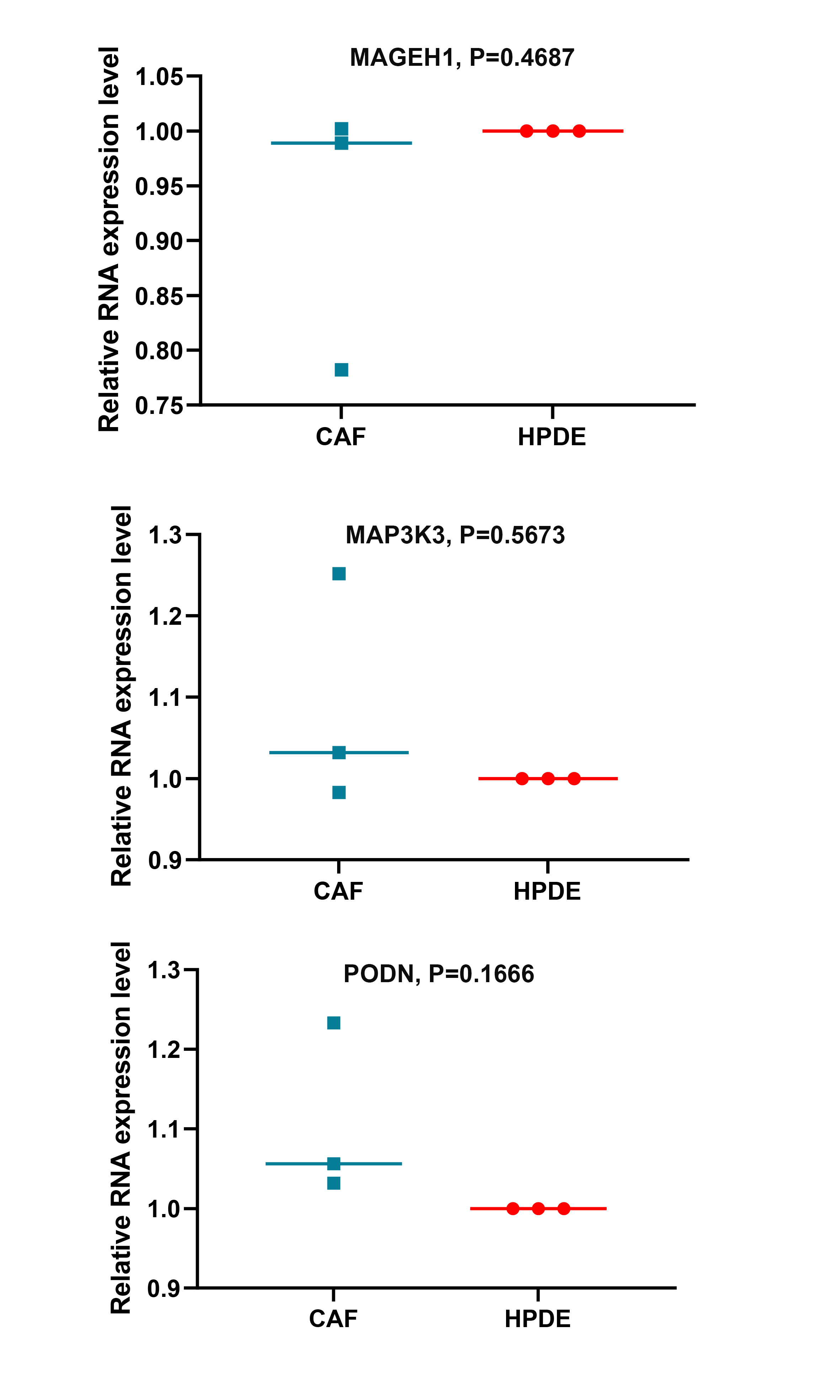

Supplement: Supplementary Figure 5 — The expression of target genes in fibroblasts derived from pancreatic cancer. [file Image_5.jpeg]
